# Optimizing raffinose family oligosaccharides content in plants: A tightrope walk

**DOI:** 10.3389/fpls.2023.1134754

**Published:** 2023-03-28

**Authors:** Rajarshi Sanyal, Sandeep Kumar, Arunava Pattanayak, Abhijit Kar, Sujit K. Bishi

**Affiliations:** ^1^ School of Genomics and Molecular Breeding, ICAR-Indian Institute of Agricultural Biotechnology, Ranchi, Jharkhand, India; ^2^ Division of Biochemistry, ICAR-Indian Agricultural Research Institute, Pusa, New Delhi, India; ^3^ Automation & Plant Engineering Division, ICAR-National Institute of Secondary Agriculture, Ranchi, Jharkhand, India

**Keywords:** abiotic stress, antinutritional factors, flatulence, raffinose, stachyose

## Abstract

Plants synthesize various compounds for their growth, metabolism, and stress mitigation, and one such group of compounds is the raffinose family of oligosaccharides (RFOs). RFOs are non-reducing oligosaccharides having galactose residues attached to a sucrose moiety. They act as carbohydrate reserves in plants, assisting in seed germination, desiccation tolerance, and biotic/abiotic stress tolerance. Although legumes are among the richest sources of dietary proteins, the direct consumption of legumes is hindered by an excess of RFOs in the edible parts of the plant, which causes flatulence in humans and monogastric animals. These opposing characteristics make RFOs manipulation a complicated tradeoff. An in-depth knowledge of the chemical composition, distribution pattern, tissue mobilization, and metabolism is required to optimize the levels of RFOs. The most recent developments in our understanding of RFOs distribution, physiological function, genetic regulation of their biosynthesis, transport, and degradation in food crops have been covered in this review. Additionally, we have suggested a few strategies that can sustainably reduce RFOs in order to solve the flatulence issue in animals. The comprehensive information in this review can be a tool for researchers to precisely control the level of RFOs in crops and create low antinutrient, nutritious food with wider consumer acceptability.

## 1 Introduction

Plants are sessile and hence must face the real challenges of nature in terms of biotic and abiotic stresses. These inevitable environmental factors often profoundly affect plant metabolism and photosynthesis, leading to a significant decline in crop yields and productivity. Plants have adapted different strategies to cope with the ever-changing climate during evolution. Plants use carbohydrates or their derivatives as stress-sensing and signalling molecules (Cummings, 2019) for coordinating metabolism with developmental features, plant growth and responses to external stimuli (Rolland et al., 2006). Furthermore, low-molecular weight soluble sugars, amino acids, and amines accumulate in the cytosol or vacuoles and help in the cell’s osmotic adjustment and also protect the cell membrane and other cell components from reactive oxygen species (ROS). Raffinose family oligosaccharides (RFOs) and Fructooligosaccharides (FOS) are one such class of soluble sugars which play an important role in abiotic stress response.

RFOs, such as raffinose, stachyose, and verbascose, are the non-reducing carbohydrates formed by α-1,6-galactosyl extensions onto the glucose moiety of sucrose. These compounds are nearly ubiquitous in crops (Minorsky, 2003), with contents varying from species to species and even from plant to plant, depending on the growing conditions. RFOs act as reserve carbohydrates (Gangl and Tenhaken, 2016) and are reported as compatible solutes that function like antioxidants, are a component of carbon partitioning strategies and may act as stress signals (Elsayed et al., 2014). RFOs benefit plants, but humans and other monogastric animals find them difficult to digest due to the absence of the enzyme required for their hydrolysis. Food containing higher RFOs takes a shorter time to pass through the digestive tract, causing reduced absorption of beneficial nutrients. The lack of hydrolysis in the small intestine and subsequent fermentation by the gut flora in the colon results in flatulence (Valentine et al., 2017). This limits the consumption of crops with higher RFOs (Kannan et al., 2018, Kasprowicz-Potocka et al., 2022). The balance between crop quality improvement and plant metabolism/immunity makes RFOs manipulation challenging for researchers.

This review highlights the structural chemistry of RFOs leading to their biosynthesis and subsequent degradation. The latest information on the genetic control of RFOs, their distribution, transport and physiological significance are also discussed. Recent studies targeting the manipulation of RFOs biosynthesis and transport to minimise their content in the human diet have also been highlighted, paving the way for producing low antinutrient, consumer-preferred food crops.

## 2 Chemical structure of RFOs and their types

**Figure 1:**
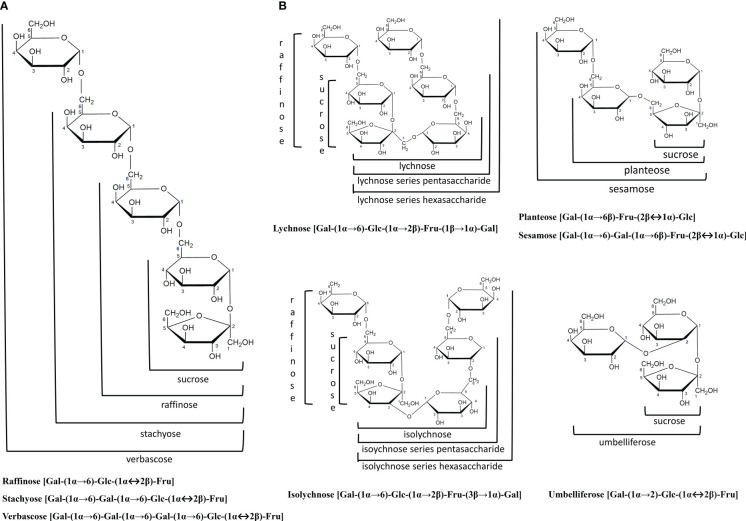
The general structure of raffinose family oligosaccharides (RFOs) and their related families. **(A)** RFOs are α(1-6) galactosyl substituted derivatives of the sucrose molecule, forming a non-reducing oligosaccharide. Raffinose, Stachyose and Verbascose are the major RFOs members in plants. **(B)** RFOs isomers with galactosidic linkages at other carbons of glucose (Umbelliferose), fructose (Planteose and Sesamose), or raffinose (Lychnose and Isolychnose). These classes do not fall under the RFOs. Structures were drawn with ChemDraw (version 12.0.2) software.

Raffinose family oligosaccharides (RFOs) are formed when galactose units are attached to the glucose moiety of sucrose *via* α-1,6-galactosidic linkages (**Figure 1A**). Galactinol serves as the galactose donor to sucrose, producing raffinose (trisaccharide), the first member of the RFOs family (Elango et al., 2022). Further addition of galactosyl residues forms stachyose (tetrasaccharide) and verbascose (pentasaccharide), which accumulate primarily in dicotyledonous seeds (Sengupta et al., 2015). Oligosaccharides with a higher degree of polymerization (DP) include ajugose (hexasaccharide), which is limited to some species of the *Lamiaceae* family, particularly *Ajuga reptans* (Haab and Keller, 2002). Higher plants seldom produce RFOs isomers with galactosidic linkages at other carbons of glucose (such as umbelliferose), fructose (such as Planteose and Sesamose), or both glucose and fructose moieties concurrently (such as Lychnose and Isolychnose) (Vanhaecke et al., 2010). However, these classes do not fall under the RFOs (**Figure 1B**). Unlike RFOs, sugars like lychnose/isolychnose are exclusively produced by the *Caryophyllaceae* family, acting as chemotaxic markers of this family (Madore, 2001).

## 3 Biosynthesis of RFOs in plants

**Figure 2:**
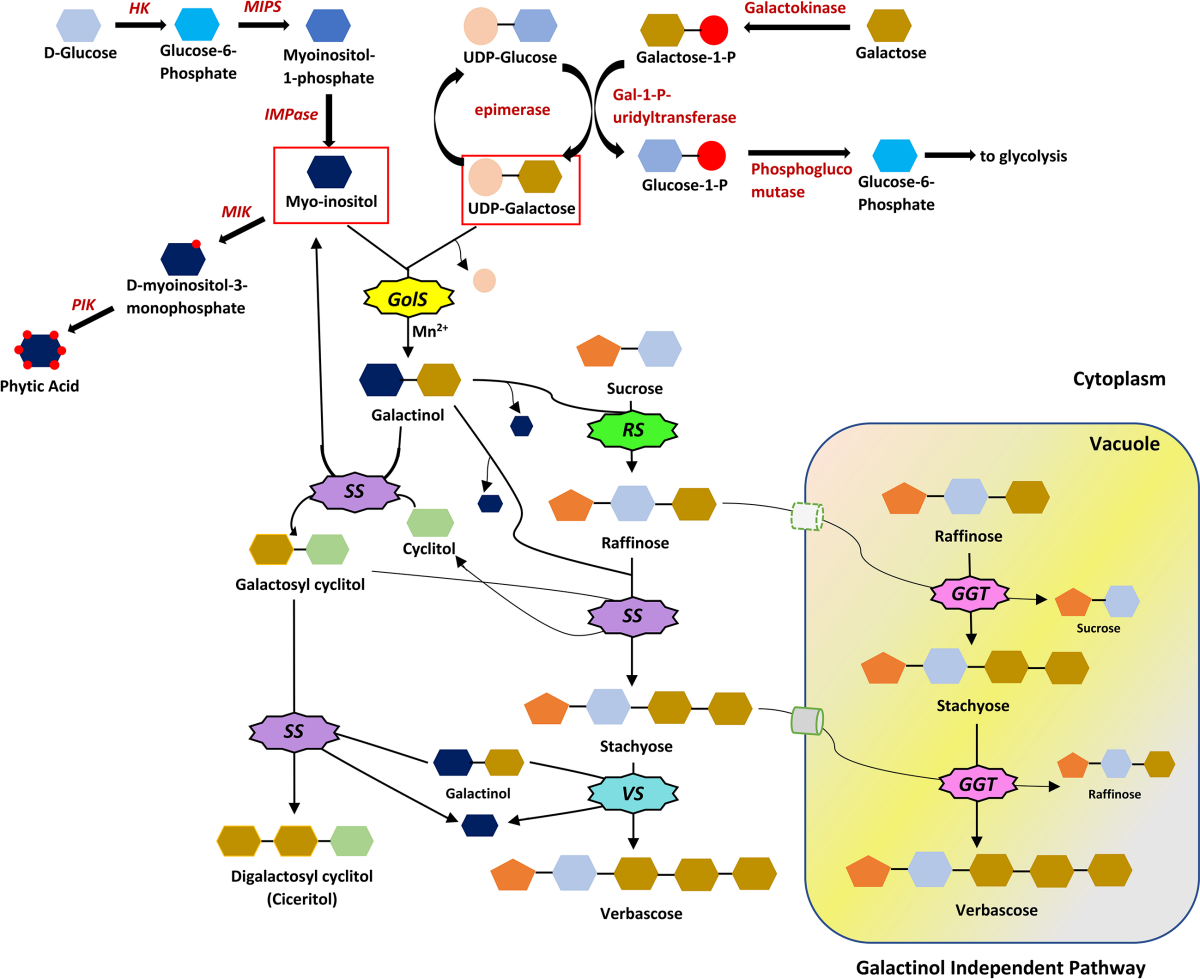
The RFOs biosynthetic pathway. Myo-inositol and UDP-Galactose serve as the precursors for galactinol. The reaction is catalysed by galactinol synthase (*GolS*) and Mn^2+^ as a cofactor. Galactinol is the galactose donor to sucrose, forming raffinose with the help of raffinose synthase (*RS*). The addition of galactose moiety to raffinose is catalysed by stachyose synthase (*SS*), producing stachyose. *SS* has a broad substrate specificity, catalyzing the production of ciceritol in some plants. Verbascose synthase (*VS*) catalyses the formation of verbascose in the galactinol-dependent pathway. A galactinol-independent pathway also exists in some crops or even in different tissues of the same crop, where galactan: galactan galactosyltransferase (*GGT*) catalyses the formation of RFOs, utilizing the galactose moiety from lower order RFOs. All the reactions are reversible in galactinol-dependent and independent pathways. *HK*, Hexokinase; *MIPS*, Myoinositol-1-Phosphate Synthase; *IMPase*, Inositol mono phosphatase; *MIK*, Myoinositol Kinase; *PIK*, Phosphoinositol Kinase.

RFOs biosynthesis begins with galactinol synthase (*GolS*), catalysing the galactosyl transfer to myo-inositol from UDP-D-galactose, synthesizing galactinol (dos Santos and Vieira, 2020). A higher concentration of galactinol than UDP-D-galactose in the developing seed suggests the function of galactinol as a transient galactosyl store, separated from primary carbohydrate metabolism (Peterbauer and Richter, 2001). Raffinose synthase (*RS*) catalyzes raffinose synthesis by a galactose-transfer (from galactinol) to glucose moiety of sucrose (Li et al., 2020). Myoinositol released in this process returns to the myoinositol pool. Raffinose synthase is said to be the most unstable enzyme in this pathway (Tian et al., 2019). Raffinose can cause a product inhibition effect on *RS*. So, it is rapidly converted to stachyose by stachyose synthase (*SS*) (Tian et al., 2019). Further, galactose transfer from galactinol to stachyose is catalyzed by verbascose synthase (*VS*), yielding verbascose (**Figure 2**). Unlike *RS*, *SS* exhibits a broad substrate specificity, using a range of galactosyl cyclitols (galactosyl ononitol, galactopinitol A, galactinol) and methylated inositols (ononitol and pinitol) or myoinositol as galactosyl donors and acceptors, respectively. (Hoch et al., 1999; Peterbauer and Richter, 2001). Galactosylation of pinitol yields galactopinitol A, which acts as a galactosyl donor (to raffinose) and acceptor (yielding ciceritol). A strong negative correlation between digalactosyl cyclitol (ciceritol) and verbascose was found in two lentil cultivars (Frias et al., 1999), as the two pathways are linked *via* STS, inhibiting each other.

**Figure 3:**
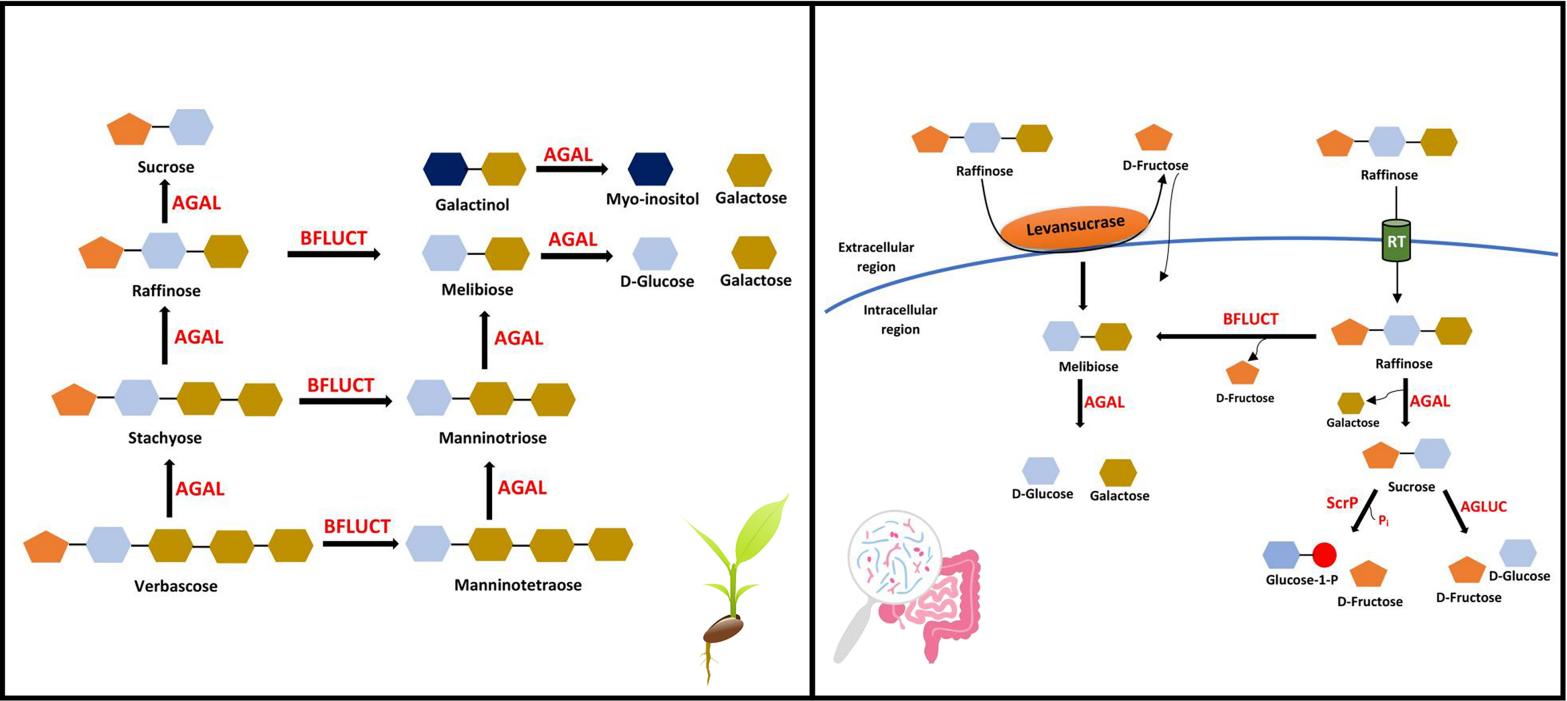
RFOs catabolism. RFOs are hydrolyzed by α-galactosidase (AGAL) and β-fructofuranosidase (BFLUCT) in most plants, producing simple sugars (left). RFOs utilization by gut microbiota in humans and animals *via* extracellular (levansucrases) or intracellular (raffinose transporter, RT) hydrolysis. AGLUC, α-glucosidases; ScrP, Sucrose Phosphorylase.

Two members of the *Lamiaceae* family (*Ajuga reptans* and *Coleus blumei*) exhibit a galactinol-independent pathway, where the galactan: galactan galactosyltransferase (*GGT*) enzyme catalyzes the galactosyl transfer from one RFO to another (Haab and Keller, 2002) (**Figure 3**). Stachyose synthesized in the cytoplasm can be transported *via* the stachyose/H^+^ antiporter to the vacuole to participate in the galactinol-independent pathway (Greutert et al., 1998). *GolS*, *RS*, *STS* being extravacuolar and stachyose, verbascose and ajugose being exclusively vacuolar, suggests the synthesis of higher-order RFOs *via* a galactinol-independent pathway in the vacuole (Peterbauer and Richter, 2001). However, in seeds, the alkaline pH (pH 6.7) of vacuoles creates an unfavorable condition for acidic *GGT* enzyme, facilitating RFOs biosynthesis in the conventional galactinol-dependent pathway (Herman and Larkins, 1999; Peterbauer and Richter, 2001; Haab and Keller, 2002).

The RFOs and phytic acid biosynthetic pathways share a common intermediate (myoinositol). Low phytic acid mutants have increased myoinositol levels, possibly contributing to RFOs accumulation (Zhawar et al., 2011; Redekar et al., 2020). Higher sucrose concentrations also increase the accumulation of raffinose (Borisjuk et al., 2002). From this, it is difficult to say which substrate (myoinositol or sucrose) is more important. Since monocots predominantly accumulate raffinose, sucrose may play an important role, but for dicots, which mainly synthesize stachyose or verbascose, galactinol (from myoinositol) play a critical role rather than sucrose. This implies that all the metabolites are tightly regulated and that any changes can markedly affect RFOs deposition in seeds (Karner et al., 2004). Further study can be conducted to establish the differences between the dicot and monocot raffinose synthase genes, causing differential accumulation of RFOs in such plants.

## 4 Genetic control of RFOs

Genes encoding enzymes involved in RFOs biosynthesis primarily have either seed-specific or phloem tissue-specific expression. Seed-specific *GolS* expression was reported to confer seed desiccation tolerance in tomatoes (Downie et al., 2003). No GolS expression in flowers, fruits, roots and endosperm, but a very high expression in leaves was observed when coffee plants were exposed to stress (drought/salt/heat) (dos Santos et al., 2011). The spatio-temporal expression patterns of hybrid poplar *GolS* homologues (Unda et al., 2012) and leaf-specific or anther-specific expression of cloned *GolS* genes from cotton (Zhou et al., 2012) suggest *GolS* expression is highly tissue-specific. *GolS1* was also reported to facilitate raffinose synthesis in the storage pool of the common bugle (*Ajuga reptans*). At the same time, *GolS2* plays a central role in synthesizing galactinol to transport the RFOs pool (Sprenger and Keller, 2000). Transcriptomic analysis of water stress-treated peanuts identified *AdGolS3* as a candidate gene for drought tolerance (Vinson et al., 2020). Similar studies on kiwifruit (*Actinidia chinensis*) identified *AcRS4* as critical during salt stress (Yang et al., 2022). The leaf and latex-specific *HbGolS1* and latex-specific expression of *HbRS1* were also reported in rubber (*Hevea brasiliensis*) (Lu et al., 2022). Among six *GolS* genes and the three *RS* genes in soybean, *GmGolS1_A*, *GmRS2_A*, and *GmRS2_B* form attractive gene targets because of their seed-specific expression patterns (de Koning et al., 2021). *GmGolS1_A* expression was highest during seed maturity, whereas soybean vegetative tissues primarily showed *GmGolS1_B* expression (Le et al., 2020). In *Arabidopsis thaliana*, among seven *GolS* isoforms, three isoforms, *AtGolS1*/*AtGolS2* and *AtGolS3* were induced during drought/salt/heat stress and cold stress, respectively (Taji et al., 2002). The inability of galactinol and raffinose accumulation in *AtGolS1* mutants also suggests *AtGolS1* as the major *GolS* isoform facilitating RFOs accumulation under heat stress (Panikulangara et al., 2004). Among six putative *RS* genes in *Arabidopsis*, overexpression of two *RS* genes showed oxidative stress tolerance in tobacco (Nishizawa et al., 2008). High seed-specific expression of *PvGolS1* and *PvRS2* in common beans (*Phaseolus vulgaris*) (de Koning et al., 2021) and *AhRS14* and *AhSS7* in peanuts (Sanyal et al., 2023) made them good candidates to knock out for low RFOs cultivar development just like the low raffinose soybean cultivars with mutated *RS2* (Dierking and Bilyeu, 2008; Bilyeu and Wiebold, 2016).

Three transcription factors (TFs) can regulate *GolS* gene expression: heat shock factors (HSFs), DREB1A/CBF3, and WRKY transcription factors (Elsayed et al., 2014). HSFs and DREB1A/CBF3 are reported in *Arabidopsis* (Panikulangara et al., 2004; Maruyama et al., 2009), while WRKY regulates both *GolS* and *RS via* W-box cis-elements present in their promoters, as explained in *Boea hygrometrica* (Wang et al., 2009). GolS1 and GolS2 expression is regulated by HSFs like *HsfA1a*, *HsfA1b*, and *HsfA2* in *Arabidopsis* (Nishizawa et al., 2008), *HsfA4a* in mustard (Lang et al., 2017) and *HsfA2* in maize (Gu et al., 2019). *HsfA2* and heat shock binding protein (*HSBP*) physically interact with each other and antagonistically modulate *GolS* expression. Overexpression of maize *ZmDREB1A* in the leaf also showed upregulation of *ZmRS* by binding to the DRE motif in the *ZmRS* promoter, enhancing raffinose synthesis and chilling stress tolerance (Han et al., 2020). A MYB-like transcription factor (AQUILO) isolated from Amur grapes (*Vitis amurensis*) improved cold tolerance through the upregulation of *GolS* and *RS* and osmoprotectant accumulation (Sun et al., 2018). Ethylene-responsive factors (ERFs) (PtrERF108) from trifoliate orange (*Poncirus trifoliata)* also target raffinose synthase *(PtrRafS*) directly, modulating raffinose levels in response to cold stress (Khan et al., 2021). Most TFs regulating RFOs in response to cold stress have been reported, while limited information is available for TF-mediated RFOs modulation in other stress situations.

The interplay of RFOs and phytohormones is also tightly controlled. Studies suggest ABA-induced RFOs accumulation in alfalfa somatic embryos (Blöchl et al., 2005) and regulation of maize *GolS2* expression by VIVIPAROUS1- ABA INSENSITIVE5 (*ZmVP1- ZmABI5*) interaction (Zhang et al., 2019). The studies observed an increase in *GolS* activity and raffinose accumulation, revealing an ABA-RFOs crosstalk, the mechanism of which is yet to be identified. Promoter-GUS study by Salvi et al., 2020, demonstrated the positive influence of ABA and dehydration stress on chickpea *GolS* (*CaGolS1 and CaGolS2*) gene. Improved chlorophyll retention, relative water content and lower H_2_O_2_, malondialdehyde (MDA) content, and ion-leakage in transgenic lines suggested the potential role of GolS in modulating ROS and alleviating dehydration stress. *OsPP65* (a type 2C protein phosphatase) knockout rice plants showed significant expression of ABA and jasmonic acid biosynthetic genes as well as their high endogenous levels during osmotic (salt) stress (Liu Q. et al., 2022). Metabolomics analysis showed higher endogenous galactose and galactinol content but a lower raffinose content in the transgenic rice suggesting negative regulation of *OsPP65* through ABA and JA-mediated modulation of RFOs during salt stress tolerance. The role of Brassinosteroid (24-epibrassinolide/EBR) in the positive regulation of tea GolS gene (*CsGolS2*) and enhancement in ABA signal transduction (Zhang et al., 2022) also suggests the possible regulation of the RFOs gene by phytohormones.

## 5 Degradation of RFOs

α-Galactosidases (*AGAL*) are activated during germination and hydrolyze RFOs into simpler molecules, i.e., sucrose and galactose (**Figure 3**). The galactokinases act upon the galactose removed during this process, forming D-galactose-1-phosphate. This compound is further metabolized by UDP-D-glucose-hexose-1-phosphate uridyltransferase (*via* the Leloir pathway) or UDP-D-galactose pyrophosphorylase (*via* the pyrophosphorylase pathway) (Peterbauer and Richter, 2001). RFOs and *AGALs* co-occur in protein storage vacuoles, but simultaneous synthesis and degradation are prevented due to the vacuole’s high pH during the reserve deposition and storage phase (Keller and Pharr, 1996). Evidence also negates the function of the protein storage vacuole as a lytic compartment (Jauh et al., 1999; Peterbauer and Richter, 2001). *AGAL* synthesised *de novo* (Group II *AGAL*) plays a role in galactomannan degradation, while pre-existing *AGAL* (Group I *AGAL*) appears to be responsible for RFOs hydrolysis (Katrolia et al., 2014). *AGALs* with acidic pH optima are also present in extracytoplasmic or vacuolar regions, but they are not effective at hydrolyzing larger RFOs, such as stachyose, and generally show a preference for small oligosaccharides (Peterbauer and Richter, 2001).

In animals, due to the lack of α-galactosidase enzyme, RFOs cannot be utilized. It passes to the lower gut and gets fermented by the gut microbiota (Arunraj et al., 2020). Out of thousands of bacteria in the human gut, about 10-15% have the potential to utilize raffinose as their substrate (Mao et al., 2018). Bacteria that prefer galactose to glucose or fructose as an energy source metabolize stachyose better than raffinose, while most bacteria commonly metabolize raffinose (Zartl et al., 2018). All bacteria that utilize raffinose do not necessarily have *AGAL* activity but still manage to degrade raffinose by using enzymes like β-fructofuranosidases (*BFLUCT*, hydrolase class) or levansucrases (transferase class). *BFLUCT* removes the fructosyl moiety of raffinose (yielding melibiose) and stachyose (yielding manninotriose). Bacteria producing both *AGAL* and *BFLUCT* can hydrolyze raffinose into galactose, glucose, and fructose. Two types of hydrolysis generally occur: intracellular and extracellular. In the case of intracellular hydrolysis, raffinose hydrolysis occurs inside the cell after transporting it *via* raffinose transporters. In contrast, for extracellular hydrolysis, raffinose can be hydrolyzed into fructose and melibiose by levansucrases (**Figure 3**). The hydrolysis products are transported inside the cell and metabolized for energy supply. Hence, glycosidases and transporters play a vital role, enabling gut bacteria to utilize galactosides differently (Teixeira et al., 2012; Mao et al., 2018). However, most studies used raffinose as the substrate for gut bacteria, while the effect of stachyose and verbascose as a substrate needs further validation.

## 6 Distribution of RFOs in crops

The content and composition of RFOs vary across the genotypes and environmental conditions (**Table 1**) (Kumar et al., 2010; Redekar et al., 2020). Seeds are the primary storage site for RFOs. Plants may store RFOs in tubers or mesophyll cells of photosynthesizing leaves, sometimes reaching even 25-80% of dry weight (Keller and Matile, 1985). All seed parts, viz., embryo, endosperm or seed coat, can retain α-galactosides at varying levels (Martínez-Villaluenga et al., 2008; Sengupta et al., 2015). Reports suggest that lupin seeds have the highest RFOs, followed by soybean (Ruiz-López et al., 2000; Martínez-Villaluenga et al., 2008). Stachyose seems to be the predominant RFO in dicot crops. However, monocot seeds such as barley and wheat primarily accumulate raffinose (Yan et al., 2022). Among the commonly cultivated crops, ajugose was found exclusively in lupin seeds. In crop plants, the reports suggest that ciceritol, a pinitol digalactoside, is found only in chickpeas and lentils, with chickpeas accumulating the maximum amount (1.2-3.1%). Among legumes, groundnut and faba bean have been reported to have lower amounts of RFOs (0.12-.076% and 1.0-4.5%, respectively) (Bishi et al., 2015; Kannan et al., 2018; Sanyal et al., 2023). Analysis of RFOs in *Brassica*, barley (Andersen et al., 2005), and wheat (Huynh et al., 2008) suggested that non-legumes contain comparatively lower amounts of these oligosaccharides. Although various studies discussed the variations in RFOs content/composition, the evolutionary reason behind the accumulation of high DP compounds (verbascose, ajugose, ciceritol), having higher energy costs, is still unexplored. Moreover, there is significant research on RFO concentrations in legume crops, while it is still scanty for non-legumes.

**Table 1:** Distribution of individual α-galactosides in commonly cultivated crops.

Genus	*Species*	Raffinose (%)	Stachyose (%)	Verbascose (%)	Ajugose (%)	Ciceritol (%)	Total RFOs (%)	References
*Pisum* (Field pea)	*sativum*	0.4–2.3	0.3–5.5	0–4.3	–	–	2.3–9.6	(Vidal-Valverde et al., 2003; Martínez-Villaluenga et al., 2008; Gawłowska et al., 2022)
*Lupinus* (Lupin)	*albus*	0.3–0.6	5.0–7.2	0–0.9	0.2–0.5	–	5.5–8.1	(Trugo et al., 1988; Ruiz-López et al., 2000; Andersen et al., 2005; Martínez-Villaluenga et al., 2008)
	*luteus*	0.5–0.6	6.1–8.6	2.8–3.5	0.6–4.6	–	11–16.1	(Trugo et al., 1988; Ruiz-López et al., 2000; Andersen et al., 2005; Martínez-Villaluenga et al., 2008)
	*angustifolius*	0.6–1.2	3.6–5.2	0.8–2.5	1.7–2.6	–	6.7–11.5	(Trugo et al., 1988; Ruiz-López et al., 2000; Andersen et al., 2005; Martínez-Villaluenga et al., 2008)
	*mutabilis*	1.9	2.3	1.0	0.2	–	5.1	(Trugo et al., 1988; Ruiz-López et al., 2000; Andersen et al., 2005; Martínez-Villaluenga et al., 2008)
*Glycine* (Soybean)	*max*	1.0–2.0	2.2–4.9	0–0.3	–	–	6.0–8.0	(Reddy et al., 1984; Naczk et al., 1997; Hollung et al., 2005; Martínez-Villaluenga et al., 2008; Bueno et al., 2018; Kannan et al., 2018)
Phaseolus	*vulgaris* (common bean)	0.2–2.5	0.2–4.2	0.1–4.0	–	–	0.4–8.0	(Reddy et al., 1984; Trugo et al., 1988; Vidal-Valverde et al., 2003; Martínez-Villaluenga et al., 2008; Zhang et al., 2019)
	*lunatus* (lima bean)	0.28-0.3	2.83-3.16	0.19-0.25	–	–	3.30-3.71	(Oboh et al., 2000; Zhang et al., 2019)
*Arachis* (Groundnut)	*hypogaea*	0.01-0.12	0.11-0.67	0-0.07	–	–	0.12-0.76	(Pattee et al., 2000; Bryant et al., 2003; Bishi et al., 2015, Sanyal et al., 2023)
*Vigna*	*unguiculata* (Cowpea)	0.41	3.22 - 4.44	0.48	–	0.04	4.15-5.37	(Elkowicz and Sosulski, 1982; Onigbinde and Akinyele, 1983; Martín-Cabrejas et al., 2008)
	*radiata* (mungbean)	0.23	0.95	1.83	–	–	3.01	(Elkowicz and Sosulski, 1982; Kannan et al., 2018)
	*mungo* (Blackgram)	trace	0.89	3.44	–	–	4.33	(Reddy et al., 1984; Kannan et al., 2018)
	*umbellate* (Ricebean)	0.05-0.2	1.18-5.77	–	–	–	1.23-5.97	(Bepary and Wadikar, 2019; Sharma et al., 2023)
*Cajanus* (Red gram)	*cajan*	0.52-0.92	0.74-1.20	3.6-6.0	–	–	4.86-8.12	(Mulimani and Devendra, 1998; Zhang et al., 2019)
*Macrotyloma* (Horsegram)	*uniflorum*	0.68	1.94	–	–	–	2.62	(Anisha and Prema, 2008; Zhang et al., 2019)
*Lens* (Lentil)	*culinaris*	0.1–1.0	1.1–4.0	0–6.4	–	0.2–2.1	1.8–7.5	(Reddy et al., 1984; Vidal-Valverde et al., 1993; Martínez-Villaluenga et al., 2008)
*Cicer* (Chickpea)	*arietinum*	0–2.4	0.4–2.6	0–4.5	–	1.2–3.1	2.0–7.6	(Reddy et al., 1984; Vidal-Valverde et al., 1993; Alajaji and El-Adawy, 2006; Martínez-Villaluenga et al., 2008; Kannan et al., 2018)
*Vicia* (Faba bean)	*faba*	0.1–1.5	0.2–2.4	1.1–2.4	–	–	1.0–4.5	(Reddy et al., 1984; Frias et al., 1999; Vidal-Valverde et al., 2003; Martínez-Villaluenga et al., 2008; Kannan et al., 2018)
*Pachyrhizus* (Yambean)	*erosus*	0.82	2.46	0.11	–	–	3.39	(Azeke et al., 2007; Zhang et al., 2019)
*Canavalia*	*ensiformis* (Jack bean*)*	0.68-0.79	0.78-0.87	3.51-3.87	–	–	4.97-5.53	(Pugalenthi and Vadivel, 2006; Zhang et al., 2019)
	*gladiata* (Sword bean*)*	0.72-1.6	0.75-2.60	3.7-6.65	–	–	5.17-10.85	(Pugalenthi and Vadivel, 2006; Zhang et al., 2019)
*Brassica*	*campestris* (Field mustard)	0.2	0.7	–	–	–	0.9	(Andersen et al., 2005; Martínez-Villaluenga et al., 2008)
	*napus* (Rapeseed)	0.2–0.4	0.7–1.7	–	–	–	0.9–2.1	(Andersen et al., 2005; Martínez-Villaluenga et al., 2008; Kannan et al., 2018)
	*nigra* (Black mustard)	0.6	1.3	–	–	–	1.9	(Andersen et al., 2005; Martínez-Villaluenga et al., 2008)
*Hordeum* (Barley)	*vulgare*	0.5	–	–	–	–	0.5	(Andersen et al., 2005; Martínez-Villaluenga et al., 2008; Kannan et al., 2018)
*Triticum* (Wheat)	*aestivum*	0.3	–	–	–	–	0.3	(Huynh et al., 2008; Kannan et al., 2018)

*values not detected have been represented by a minus (–) sign.

## 7 RFOs transport in plants

Plants belonging to Gamalei’s “Type 1 category” (such as cucurbits) have a high plasmodesmata abundance between companion cells and mesophyll cells and assimilate is loaded *via* a polymer trap mechanism in a symplastic route (Gamalei, 1989). RFOs are predominantly transported in such plants. On the other hand, “Type 2 plants” with lower plasmodesmata frequency (such as potato and *Arabidopsis*) primarily transport sucrose *via* proton symport in an apoplastic route (Turgeon, 1996; Haritatos et al., 2017). The polymer trap model states that the specialized companion cells (intermediary cells) in the minor veins are where RFOs biosynthetic enzymes transform the sucrose generated by photosynthesis in mesophyll cells (source) into RFOs (mainly raffinose and stachyose) (Cao et al., 2013). The plasmodesmata in RFOs-utilizing plants are characteristically branched on the side of companion cells, significantly reducing the plasmodesmatal pore size. The RFOs cannot diffuse back to the mesophyll (source) because they are larger and are trapped in the intermediary cells. Conversion of sucrose into RFOs favors passive entry of sucrose, while RFOs accumulation increases osmotic pressure. This makes it easier for the RFOs to migrate toward the sieve elements, followed by transportation to other areas of the plant (sinks), where *AGAL* may break them down (Turgeon and Medville, 2004). Plants can maintain a high phloem sugar concentration by producing RFOs in the intermediate cells. Although species-specific, this paradigm is primarily observed in the *Cucurbitaceae* family. Meagre amounts of RFOs are transported by Type 1 plant species lacking intermediate cells, and they mostly load assimilates *via* the apoplastic pathway (Hannah et al., 2006).

In leaves that transport, as well as store RFOs, such as *Xerosicyos danguyi* (Cucurbitaceae) and *Ajuga reptans* (Lamiaceae), RFOs biosynthetic components are present in both phloem and mesophyll tissues in different isoforms, involving complex cellular partitioning (Madore, 2001). Plants having variegated leaves (such as *Coleus blumei Benth)* do not use *AGAL* for RFOs degradation in non-photosynthesizing patches. Instead, they possibly use the reverse (or backward) reaction of *RS* and *SS* to degrade RFOs into disaccharides (galactinol and sucrose). The products obtained thereof can support respiration in the absence of photosynthesis (Madore, 2001). ^14^C-labelling study in *Cucumis blumei* indicates limited phloem transport of galactinol and efficient retention and transportation of sucrose, raffinose and stachyose. Studies estimating raffinose and galactinol levels observed 30 times lower raffinose in *Cucumis* leaves but only two-fold lower raffinose in phloem exudates (Ayre et al., 2003; Hannah et al., 2006), suggesting higher transport efficiency of raffinose as compared to galactinol.

Additionally, raffinose can supplement sucrose as phloem-mobile forms of carbon, delivering 1.5 times more carbon than sucrose at the same osmotic cost. This is often seen to support non-photosynthetic tissues and organs (Madore, 2001). When plants with an apoplastic phloem loading strategy (Type 2 plants) were engineered to follow a symplastic route (polymer trap mechanism) *via* metabolic engineering of RFOs biosynthetic genes, the synthesis of RFOs and their transportation was deficient despite the high sucrose concentration (Hannah et al., 2006; Cao et al., 2013). Theoretically, apoplasmic loaders should synthesize RFOs efficiently due to ample carbon (reduced form) in the companion cell cytoplasm where RFOs synthesis occurs. Moreover, there are no limitations in the plasmodesmatal pore size. The inability of high RFOs accumulation in companion cells can be a biochemical limitation or a cell biology problem (Yadav et al., 2015). There can be limitations in the flux of early RFOs precursors such as UDP-D-galactose and myoinositol or variations in the internal membrane and vacuoles. “Type 1” plants with numerous, highly branched plasmodesmata generally have small vacuoles, extensive endomembrane systems and companion cells larger than “Type 2” plants. Thus, the internal structure can also contribute to the biosynthesis of RFOs in companion cells (Turgeon et al., 2001). The stability, localization and interaction of enzymes with other cellular components can probably explain the inefficient synthesis and transport of RFOs in “Type 2” plants.

## 8 Physiological significance of RFOs in plants and animals

### 8.1 Seed desiccation tolerance

Under water deficit conditions, the hydroxyl groups of RFOs provide the hydrophilic interaction needed for cellular membrane and protein stabilization. A higher RFOs concentration prevents sucrose crystallization during desiccation and facilitates stable vitreous/glassy state formation (Leopold et al., 1994; Elango et al., 2022). The concentrated, highly viscous solid crystals formed within cells (intracellular glass) protect the desiccating seeds by providing stability during dormancy (Koster et al., 1991). Moreover, raffinose and its higher homologues stabilize the membrane bilayer by inserting themselves within the lipid headgroups during stress (Hincha et al., 2003). Delayed acquisition of desiccation tolerance was observed in RFOs biosynthesis mutants (*gos1, gos2* and *rs5*) of *Arabidopsis.* In contrast, the corresponding overexpression lines exhibited higher RFOs and enhanced desiccation tolerance (Jing et al., 2018). Relatively high level of reducing sugars in green chickpea pods and their subsequent reduction from yellow pod stage through post-germination stage, indicated a continual supply of reducing sugars for the seed’s energy requirements as it dries out, preparing it for desiccation and germination (Arunraj et al., 2020). Experiments also demonstrated the raffinose-mediated increase in antioxidant gene expression and their stabilization (Nishizawa et al., 2008, Salvi et al., 2016, Keller et al., 2021), facilitating ROS detoxification. These experiments suggest probable mechanisms protecting the seed and helping them remain viable in the dry state.

### 8.2 Abiotic stress tolerance

**Figure 4:**
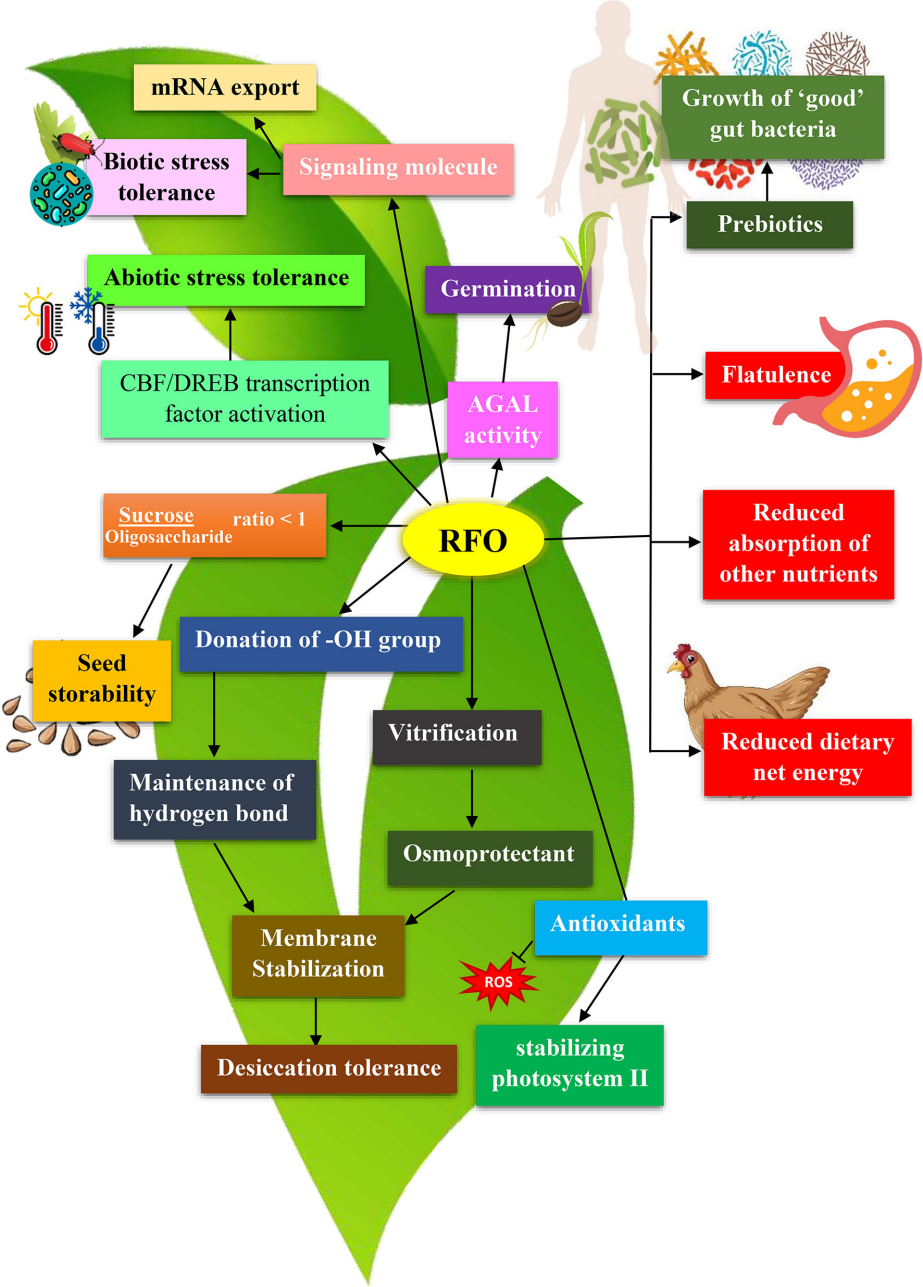
Functions of Raffinose Family Oligosaccharides (RFOs) in plants and animals. RFOs in plants provide abiotic stress tolerance, serve as a storage compound upon seed germination, maintain osmotic balance, help seed storability, and stabilize membranes. In human and monogastric animals, these same RFOs are responsible for flatulence production, reduced net dietary energy and decreased absorption of other nutrients. However, when RFOs reach the colon, they serve as prebiotics and help in the growth of probiotic microorganisms.

Various molecular and biochemical changes occur during the acclimatization of plants to cold and drought (**Figure 4**). A prominent pathway that transcriptionally induces target gene expression involves cold-responsive element-binding factor/dehydration-responsive element-binding factor CBF/DREB (Jaglo et al., 2001; Lata and Prasad, 2011). During cold acclimation, the transcription factor CBF3 is overexpressed, which induces the accumulation of osmoprotective substances, including RFOs, in *Arabidopsis* (Gilmour et al., 2000). *GolS* activity also increased under cold exposure in kidney bean seeds (Liu et al., 1998) and tomato leaves (Downie et al., 2003; Nishizawa et al., 2008). Among the seven *GolS* members of *Arabidopsis*, *GolS1* and *GolS2* mRNAs were expressed in leaf during drought stress and salt stress, while *GolS3* was induced during cold stress (Taji et al., 2002; Elsayed et al., 2014). *PhGolS1-1* was recognized as a direct target of *PhZFP1*, a C2H2-type zinc finger protein, modulating galactinol synthesis and contributing to cold tolerance in *Petunia hybrida* (Zhang et al., 2022). Increased cold tolerance as a result of raffinose accumulation has also been reported in maize (Han et al., 2020), trifoliate orange (*Poncirus trifoliata L.*) (Khan et al., 2021), and barrel clover (*Medicago truncatula*) (Sun et al., 2021), where *RS* and *GolS* have been proposed to be the primary target of the associated transcription factors (HSFs, DREB1A/CBF3 and WRKY). Additionally, *GolS1* was identified as a heat shock factor (HSF)-dependent gene of *Arabidopsis* involved in vegetative tissue-specific osmolyte synthesis during stress (Panikulangara et al., 2004; Schramm et al., 2006; Nishizawa et al., 2008). Increased heat stress tolerance was observed in *Arabidopsis* plants overexpressing maize *GolS* gene (*ZmGolS2*) with increased raffinose and galactinol levels (Gu et al., 2016), while overexpression of the maize heat shock binding protein (*ZmHSBP2*) in *Arabidopsis* decreased stress tolerance due to reduced expression of RFOs genes (*AtGolS1*, *AtGolS2*, and *AtRS5*) (Gu et al., 2019). Increased RFOs content due to overexpression of *GolS* and *RS* has also been reported to improve drought tolerance in cucumber (Ma et al., 2021) and *Arabidopsis* (Li et al., 2020). Recent studies also reported higher galactinol content and increased raffinose catabolism in a type 2C phosphatase protein (*Os*PP65) knockout line of rice, conferring osmotic and salt stress tolerance (Liu et al., 2022b). Studies on the role of RFOs in alleviating abiotic stresses are ever-increasing, and the differential accumulation of galactinol and raffinose can further be studied. An opposite trend observed in raffinose levels in other instances (Le et al., 2020; Liu et al., 2022b) hints towards an additional mechanism for regulating raffinose biosynthesis.

### 8.3 Seed germination, storage, and plant development

During seed germination, the expression of *AGAL* increases, utilizing the stored RFOs as a carbon source (Martínez-Villaluenga et al., 2008). A recent report demonstrated the increase in the activity of *AGAL* during early germination and seed maturation in chickpeas (*Cicer arietinum*) (Arunraj et al., 2020). An inhibitor of α-galactosidase (1-deoxygalactonojirimycin, DGJ) in germinating pea seeds blocked RFOs breakdown and reduced germination rates to approximately 25% of control, two days after imbibition (Blöchl et al., 2007). Soybean seeds similarly experienced a delay in germination, but the germination rate was not significantly reduced, indicating a secondary role of RFOs rather than a primary one (Dierking and Bilyeu, 2008; Li et al., 2020). The positive correlation of raffinose (Yan et al., 2018) and the negative correlation of galactose (Chen et al., 2022) with seed germination in hybrid rice under natural ageing conditions also suggested the role of RFOs in seed vigour and longevity. Decreased seed longevity was also observed in maize plants with low raffinose (reduced *ZmRS* expression) (Han et al., 2020) and *Arabidopsis* plants overexpressing *AGAL* (*ZmAGA1*) (Zhang et al., 2021). The seed germination, however, was higher in low raffinose *Arabidopsis* plants (Zhang et al., 2021). Like phytic acid (phosphate reservoir), RFOs act as galactose reservoirs, interacting with PIF transcription factors to control temperature- and light-dependent germination (Gangl and Tenhaken, 2016). Early studies already reported the influence of sucrose to total oligosaccharide ratio on seed storability. A ratio less than 1 confers seed storability with a half viability period (t_1/2_) >10 years, while a ratio greater than 1 negatively impacts seed storability with a t_1/2_ <10 years (Horbowicz and Obendorf, 1994). Accumulation of soluble sugars or alcohols possibly plays a protective role by minimizing harmful effects of ROS (Salvi et al., 2022). These metabolites in high concentration can stabilize the enzymes (Ascorbate, Glutathione) involved in ROS detoxification and they also exhibit higher second-order rate constants for detoxification as compared to common antioxidants (Nishizawa et al., 2008). RFOs can react with ROS using mechanisms similar to fructans (Keller et al., 2021). resulting in the formation of sugar–phenol compounds, higher DP-neutral carbohydrates, or phenolics (Peshev et al., 2013). This mechanism can protect ROS-mediated lipid peroxidation in the tonoplast. In a recent study, Keller et al., 2021, showed ROS production in sugar beet pith tissue due to frost exposure to induce raffinose synthase gene (*BvRS5*) expression and raffinose levels in the tissue. Previous studies on transgenic *Arabidopsis* also showed overexpression of galactinol synthase (*GolS1, GolS2, GolS4*) and raffinose synthase (*RS2*) along with increased ROS-scavenging/oxidative stress tolerance (Nishizawa et al., 2008). Decreased seed vigour in maize *RS* mutants and enhanced seed vigour in *Arabidopsis* lines overexpressing *ZmRS, ZmGolS* or *AtSS* have also been reported (Li et al., 2017). These pieces of evidence suggest a potential role of RFOs in supporting seed vigour and longevity *via* ROS modulation.

Reports on RFOs influencing plant growth/development are also increasing (Unda et al., 2017; Hua et al., 2021; Liu et al., 2022a). Galactinol synthase (*AtGolS3*) overexpressing poplar plants exhibited higher lignin and cellulose deposition with increased vessels (Unda et al., 2017). *AGAL* overexpressing cucumber plants also had increased fruit vasculature and size, while its RNAi lines exhibited delayed fruit development and altered sugar metabolism (Hua et al., 2021). Reduced photosynthesis and fewer plasmodesmata decreased phloem loading in *AGAL*-silenced cucumber plants (*CsAGA2*), and the opposite trend in *CsAGA2*-overexpression lines further validated the role of *AGAL* in increasing fruit size (Liu et al., 2022a).

### 8.4 Induced systemic resistance

Plants have evolved various defence strategies with the evolution of pathogens and pests. Galactinol, raffinose and melibiose (a raffinose degradation product) induce systemic resistance to phytopathogens. Experiments on rhizobacterium *Pseudomonas chlororaphis* O6 colonization in cucumber showed an increase in endogenous galactinol levels within the leaves and conferred resistance to bacterial pathogens (*P. syringae and Erwinia carotovora*) and the leaf spot fungus *Corynespora cassiicola* (Ryu et al., 2007; Kim et al., 2008; Cho et al., 2010). Such events in plants are referred to as “sugar-based resistance” or “sweet immunity” (Gómez-Ariza et al., 2007; Bolouri Moghaddam and Van Den Ende, 2013; Morkunas and Ratajczak, 2014). Recent reports also suggest RFOs play a protective role at the initial stages of root nematode infection, but nematodes hijack them as carbon nutrients at later stages of infection (Wang et al., 2022). The study reported increased galactinol, raffinose and stachyose content with overexpression of *RS* gene at the early infection stage, followed by reduced transcript levels of *GolS*, *RS* and *STS*, reduced RFOs levels and increased *AGAL* activity during the late infection stage (Wang et al., 2022). *GolS* and *RS* overexpressing poplar plants exhibited resistance to leaf rust due to higher galactinol and raffinose levels, but *GolS* silenced lines exhibited higher disease incidence (La Mantia et al., 2018). An altered translocation stream was also observed in *Arabidopsis* when RFOs biosynthetic enzymes were expressed in ordinary companion cells, which resulted in reduced fecundity of aphid feeding (Cao et al., 2013). Aphids preferred sucrose-translocating plants over RFOs-translocating plants (Hewer et al., 2010), which presents a clue about the role of RFOs from an ecological viewpoint. Such studies can further be developed to understand the role of RFOs as a phloem mobile metabolite, supporting plant immunity.

## 9 Antinutritional effects of RFOs

### 9.1 Flatulence-inducing role

Humans and other monogastric animals cannot digest RFOs because their intestinal mucosa lacks the hydrolyzing enzyme *AGAL* (Rackis, 1975). RFOs pass down to the lower intestinal tract, where the colon microflora metabolizes them *via* anaerobic fermentation, producing excess carbon dioxide, hydrogen, traces of short-chain fatty acids (SCFAs) and methane (Minorsky, 2003; Sanyal and Bishi, 2021). Flatus accumulation in the gastrointestinal tract causes abdominal rumblings, diarrhea, cramps, pain and discomfort, deterring people from consuming high RFOs food crops (Sasi et al., 2022).

### 9.2 Interference with nutrient absorption and reduction in true metabolic energy

RFOs cause the quick passage of animal feed through the digestive system, negatively affecting the absorption of other nutrients (**Figure 4**) (Valentine et al., 2017). Improved amino acid digestion by RFO-extracted lupin feed has been observed in swine (van Barneveld, 1999). RFOs create an imbalance in the small intestine’s osmotic pressure, which reduces its absorption capacity for glucose, water, and methionine (Peterbauer et al., 2001; Martínez-Villaluenga et al., 2008). Studies have also supported the assumption that RFOs from lupin reduce nutritional value by reducing protein digestibility (Glencross et al., 2003; Martínez-Villaluenga et al., 2008). Animals fed on an RFO-rich diet see a drop in true metabolic energy (TME) due to extensive fermentation in the large intestine (Coon et al., 1990; Zhu et al., 2020; Jiang et al., 2022). TME is the net energy available for metabolism after excluding the energy lost (in urine, faeces and combustible gases) from the gross energy (Lattimer and Haub, 2010). Improvements were observed when feeding was supplemented with exogenous α-galactosidase (Jang et al., 2019; Llamas-Moya et al., 2021) or silencing the raffinose synthase gene in the food crop (Valentine et al., 2017).

Despite the negative influence of RFOs on human health, recent studies have identified some prebiotic potential of RFOs (mainly raffinose). Prebiotics stimulate calcium, magnesium and iron absorption, regulate lipid metabolism, and help modulation of immune response (Anggraeni, 2022; Kumar et al., 2022). RFOs function as prebiotics, stimulating the growth or activity of good gut bacteria (Zartl et al., 2018; Amorim et al., 2020). RFOs increased the number of Lactobacillus (beneficial bacteria) present in the vaginal microbiota (Collins et al., 2018) and decreased the pathogenic *Proteobacteria*, which causes GI tract diseases, during fermentation in the human gut (Amorim et al., 2020). Studies on 21-day-old broilers showed increased growth, cecal microbiota and gut health with enhanced immune responses after in-ovo inoculation of *B. subtilis*, raffinose, and symbiotic (Shehata et al., 2022). The beneficial influence of raffinose on gut microbiota is reviewed elsewhere (Anggraeni, 2022; Bamigbade et al., 2022). However, as mentioned in section 5, most of these studies used raffinose as a substrate, while dicots (especially legumes) generally possess higher RFOs (stachyose, verbascose), which needs consideration. The role of stachyose or verbascose as a substrate can shed light towards the actual potential of the gut microbiota in degrading RFOs.

From the above discussion, it becomes clear that RFOs benefit plant growth and development. However, their adverse effects on humans and monogastric animals require their reduction to an acceptable limit. Such an approach can preserve normal plant growth while reducing flatus production to promote human consumption and animal feed for monogastric animals like pigs and sheep.

## 10 Strategies to reduce RFOs content in plants for nutritional enhancement

### 10.1 Upregulation of α-galactosidase and enhancing galactosyl cyclitols synthesis

α-Galactosidase hydrolyses the α(1→6) linkage to break RFOs. Overexpression of *AGAL* from coffee reduced the total RFOs in peas (Polowick et al., 2009). Other RFOs degradative enzymes such as levansucrases and β-fructofuranosidases (*BFLUCT*) can also be targeted. Activation of *AGAL* after harvesting can be an interesting strategy to reduce RFOs without impacting plant development. *AGAL* from a thermophilic bacterium (e.g., *Thermotoga neapolitana*) can be transferred into grain legumes, only to be induced during canning (Wang et al., 2003; Kannan et al., 2018). An alternative strategy to reduce the RFOs concentration is increasing galactosyl cyclitol (e.g., ciceritol) synthesis (Frias et al., 1999; Peterbauer et al., 2001; Kannan et al., 2018). Ciceritol can maintain the α–galactoside activity necessary for the plants, but decrease their flatus potential, as it is hydrolyzed slower than RFOs by α–galactosidase. The stachyose synthase gene, representing a connection between RFOs and galactosyl cyclitol pathways, could be targeted in such situations.

### 10.2 Downregulation of key biosynthetic enzymes

Reducing the expression by knockdown or knockout of critical biosynthetic enzymes (*GolS, RS, SS*) can be an excellent strategy to minimise RFOs accumulation. Regulating myoinositol synthesis by suppressing myo-inositol phosphate synthase (*MIPS*) expression can also be a potential strategy (Greutert et al., 1998; Hitz et al., 2002; Ma et al., 2005). However, myoinositol is also required for various other functions, such as membrane biogenesis, light responses, receptor cycling, phosphate accumulation and mineral nutrient storage, auxin physiology, fertilization, senescence signalling, and abiotic stress response (Sengupta et al., 2015). Various approaches, such as antisense RNA technology (Greutert et al., 1998; Bock et al., 2009), RNAi approaches (Valentine et al., 2017) and CRISPR/Cas9 technology (Le et al., 2020), have recently been used to downregulate RFOs biosynthetic enzymes (**Table 2**). According to most studies, out of the major targets (sucrose concentration, myoinositol concentration, *GolS, RS, SS*), GolS is the most preferred candidate, as it commits galactose towards RFOs biosynthesis (Gangola, 2014; Kannan et al., 2018).

**Table 2:** Reports on various strategies used for reducing raffinose family oligosaccharides (RFOs) in plants.

Strategy	Crop	RFOs reduction		Reference
		Raffinose	Stachyose	
Molecular approaches				
MIPS suppression by antisense RNA approach	Potato (*Solanum tuberosum*)	12%	Galactinol (5%)	(Keller et al., 1998)
Upregulation of α-galactosidase	Pea (*Pisum sativum*)	40%	40%	(Polowick et al., 2009)
Downregulation of *GolS* by antisense approach	Canola (*Brassica napus*)	Galactinol (19-39%)	36%	(Bock et al., 2009)
RNAi construct targeting Raffinose Synthase 2	Soybean (*Glycine max*)	17%	32%	(Valentine et al., 2017)
CRISPR/Cas9 mediated *GolS* knockout	Soybean (*Glycine max*)	41.7% increase	34.1%	(Le et al., 2020)
Mapping and breeding				
**Crop**	**Observation**	**Result**		**Reference**
Pea (*Pisum sativum*)	Identification of variant *SS* gene	Production of low verbascose genotype		(Peterbauer et al., 2003)
Soybean (*Glycine max*)	Identification of independent mutant allele of the *RS2* gene	development of low RFOs line		(Dierking and Bilyeu, 2008)
	Identification of a 33 bp deletion mutant in *SS* gene	development of ultralow stachyose content (0.5%) line		(Qiu et al., 2015)
	development of an indel marker associated with low stachyose content			
	novel missense mutation in *RS3* gene along with the *RS2* gene	Development of ultralow RFOs line (Raf = 0% and Sta = 0.1%)		(Hagely et al., 2020)

### 10.3 Redirecting central carbon metabolism

Redirection of carbons involved in RFOs biosynthesis to oil or protein can be a good strategy. It has been hypothesized that carbon derived from lipid and protein turnover contributes to RFOs synthesis during the late seed maturation stage. A 10-15% reduction in lipids coincides with RFOs accumulation during seed maturity (Moretti et al., 2020). A protracted buildup of lipids without a reduction in protein content was also seen in recent research employing fast neutron-mutagenized soybean populations with deletions in genes involved in the central carbon metabolism (Kambhampati et al., 2019).

### 10.4 Mapping and breeding

Transgenics require high energy, more time, money and different regulations depending on the country, which makes the varietal release a cumbersome process, especially for feed and food purposes. Plant breeding presents a good alternative in such cases (Kannan et al., 2018). Soybean lines with high sucrose and low RFOs have been developed *via* germplasm screening and chemical mutagenesis (Hitz et al., 2002; Kannan et al., 2018), which enhanced the possibility of introgressing low RFOs phenotypes into elite genetic backgrounds (Kannan et al., 2018; Hagely et al., 2020). Studies found increased sucrose levels in low RFOs lines (Hitz et al., 2002), the genetic basis of which was associated with a deletion mutation (deletion of 331^st^ tryptophan residue) in the highly conserved coding sequence of the raffinose synthase (*RS2*) (Dierking and Bilyeu, 2008; Jo et al., 2019). Using the reverse genetics approach, a missense mutation (T107I) in the *RS2* gene was identified in soybean (Dierking and Bilyeu, 2009), and an additional mutation in *RS3* was also reported to be associated with ultralow RFOs lines (Hagely et al., 2020) (**Table 2**). Recurrent selection and traditional plant breeding methods resulted in the development of ultralow RFOs (UL RFO) phenotype (seed raffinose and stachyose content < 0.15% and < 0.54%, respectively) (Hagely et al., 2020). Association studies on indel markers with low stachyose content (Qiu et al., 2015) and genotype/environment-modulated carbohydrate profile (Bilyeu and Wiebold, 2016; Jo et al., 2019) are also available. With accelerated genomic sequencing of legumes (Das and Parida, 2013), molecular breeding is emerging as an attractive strategy (Kannan et al., 2018). It is crucial to remember that RFOs have various vital roles in plants; reducing them completely will take a toll on plant survival and yield. Gene targets with minimum hindrance to plant development and growth should be selected, and targeting seed-specific RFOs genes can be promising (de Koning et al., 2021).

## 11 Conclusion and future prospects

In recent decades, considerable progress has been made in understanding the RFOs structural diversity, biosynthesis, translocation, and catabolism. The varied roles of RFOs in plants and animals ask for the optimization of RFOs level to reduce flatulence production without interfering with the normal metabolism of the crop. Such ideal levels need to be ascertained. In the era of global warming, RFOs have the potential to enhance sugar export to phloem and improve crop performance under elevated carbon dioxide. Superimposition of the polymer trap mechanism on apoplastic phloem loaders or vice versa can be an attractive strategy to increase the economic yield of a crop. Sink-specific expression or catabolism of RFOs can modulate the hydrostatic pressure, allowing for a targeted partitioning of photoassimilates.

In future, the connection of RFOs with phytic acid, fructooligosaccharide phenols and methyl ether derivatives of cyclitols can be studied in detail so that the metabolic shift of high phytate crops into RFOs can be engineered. This will facilitate the reduction of antinutrients in crops and a limited increase in RFOs incapable of causing flatulence. Low RFOs lines with increased protein or oil content can also be prepared by altering central carbon metabolism so that they can be used in the vegetable oil industry. Moreover, using several α-galactosidase crude preparations can enhance the nutritional quality of high RFOs crops and fulfil the protein requirement of the community.

## Author contributions

RS and SB compiled and wrote the manuscript. SK, AP, and AK provided revisions of scientific content. All authors contributed to the article and approved the submitted version.
